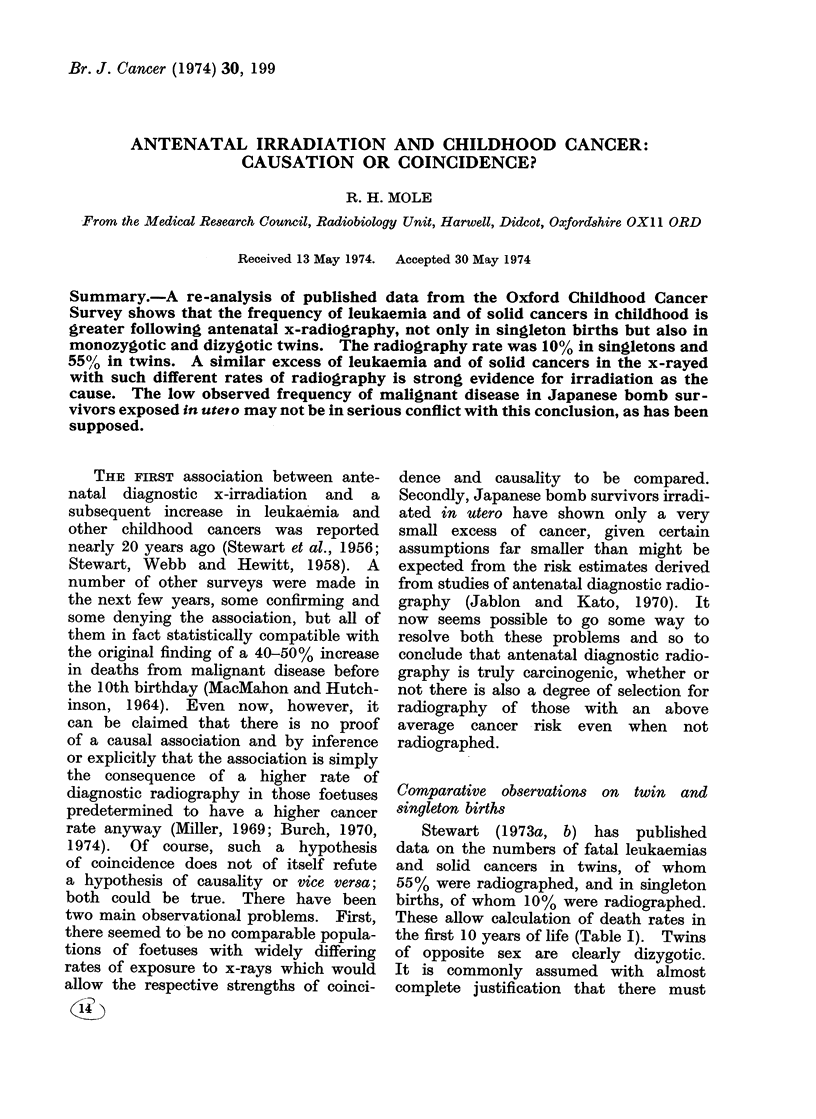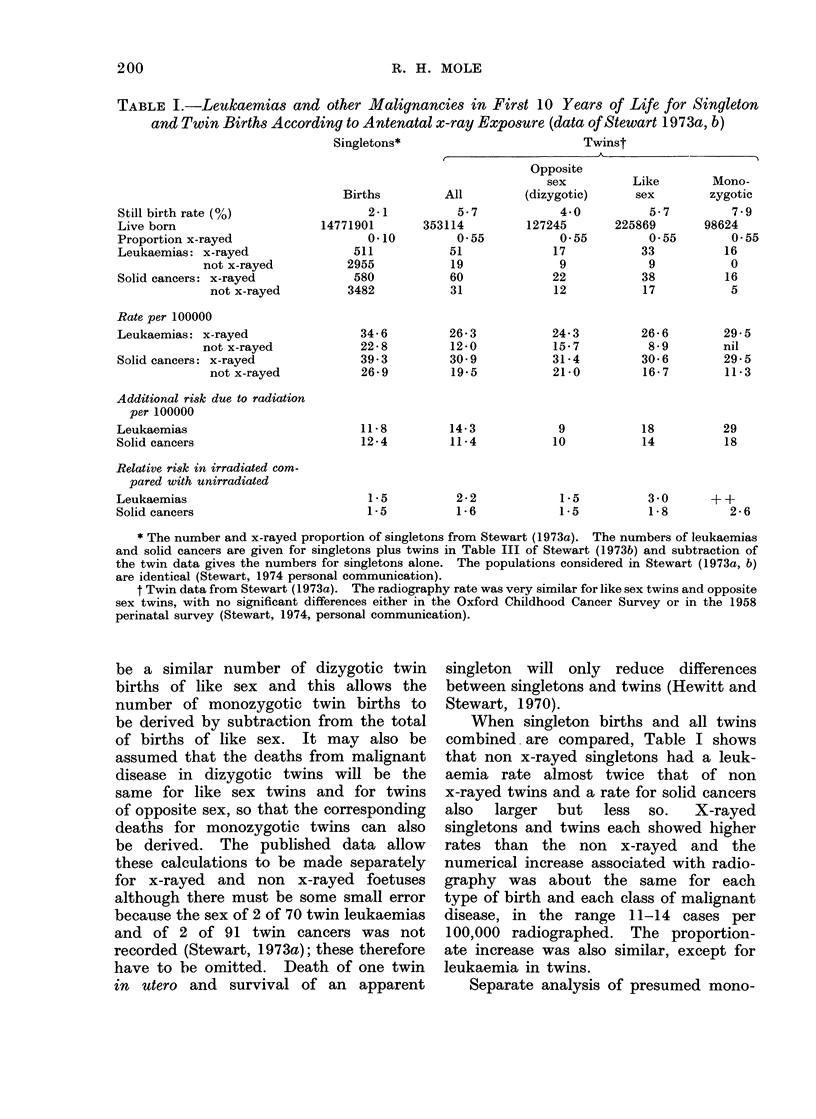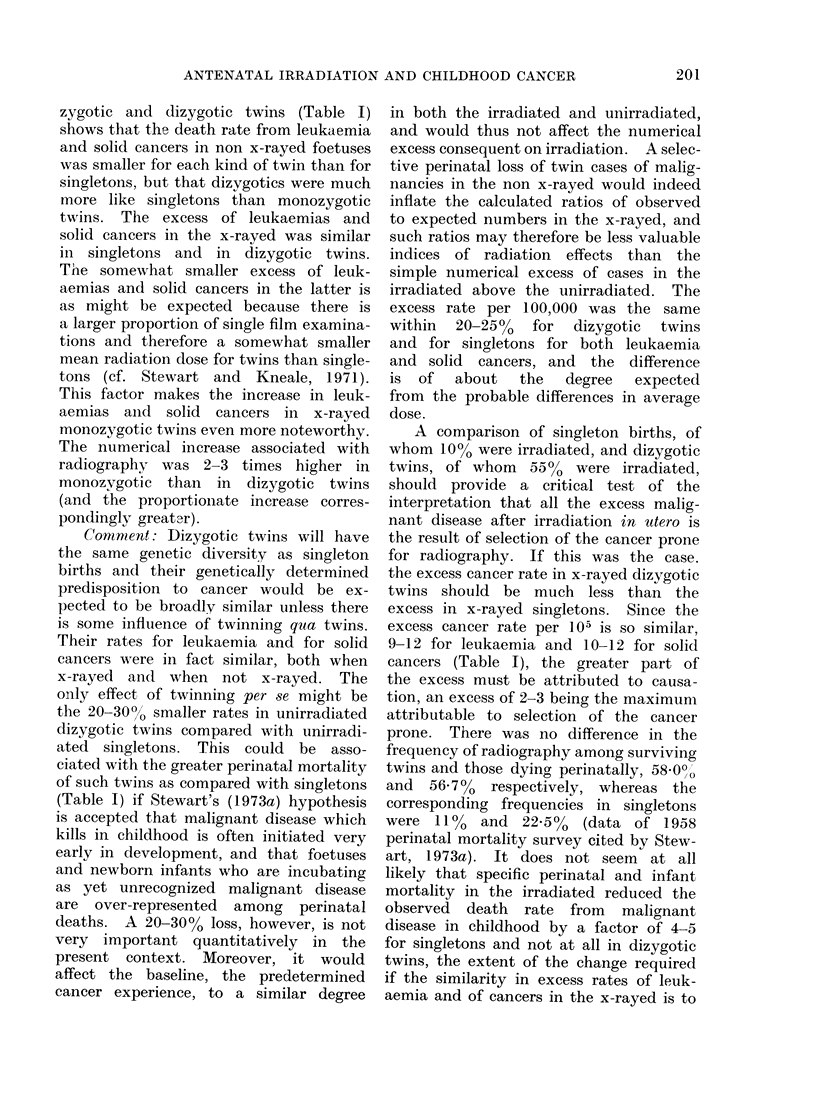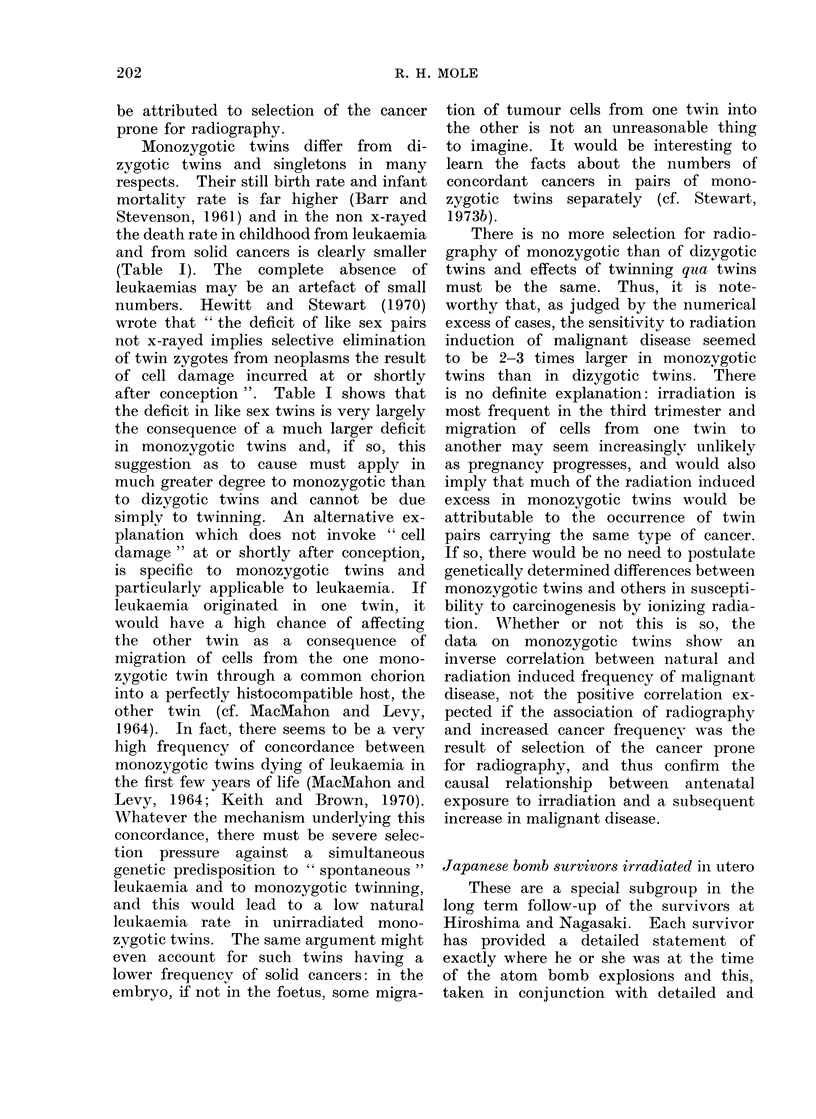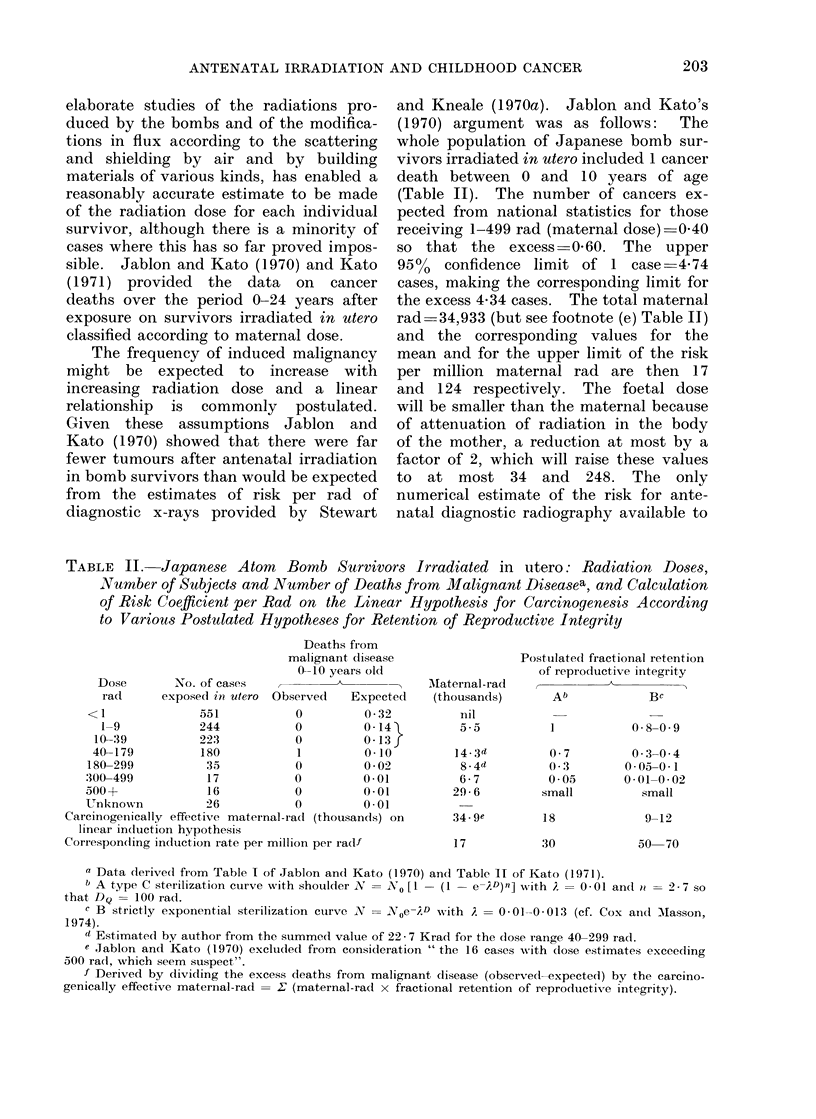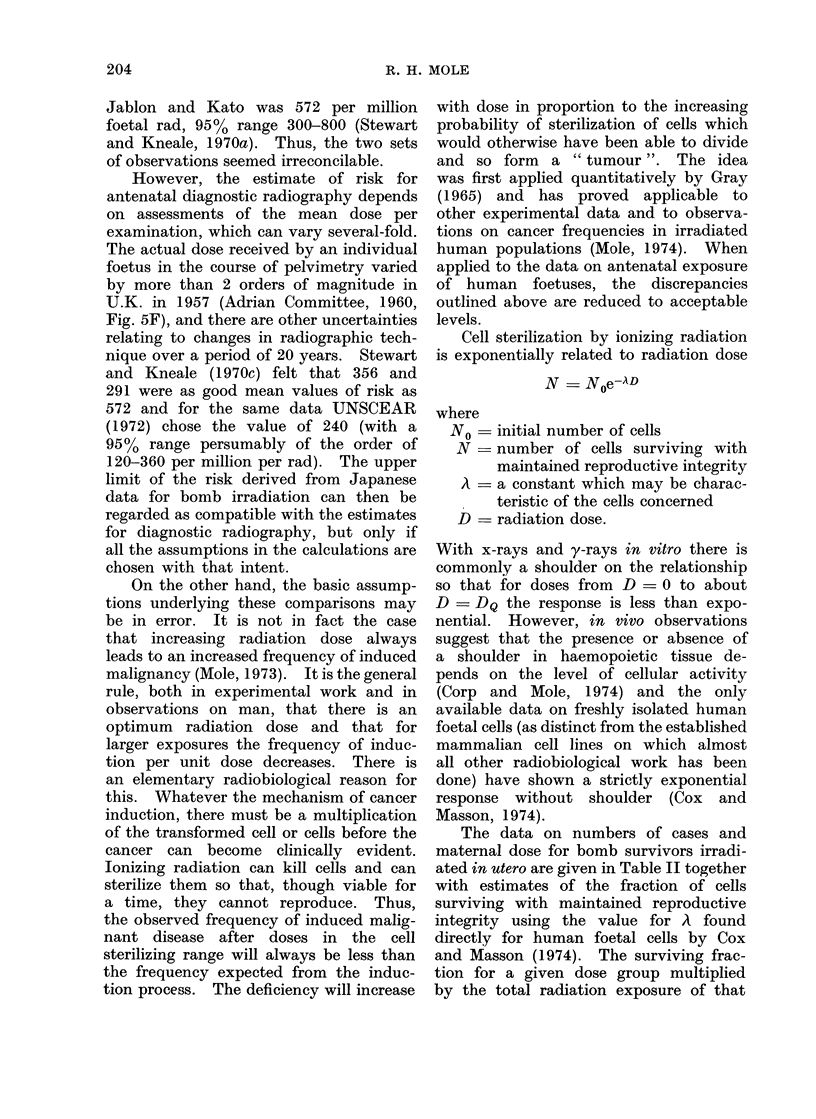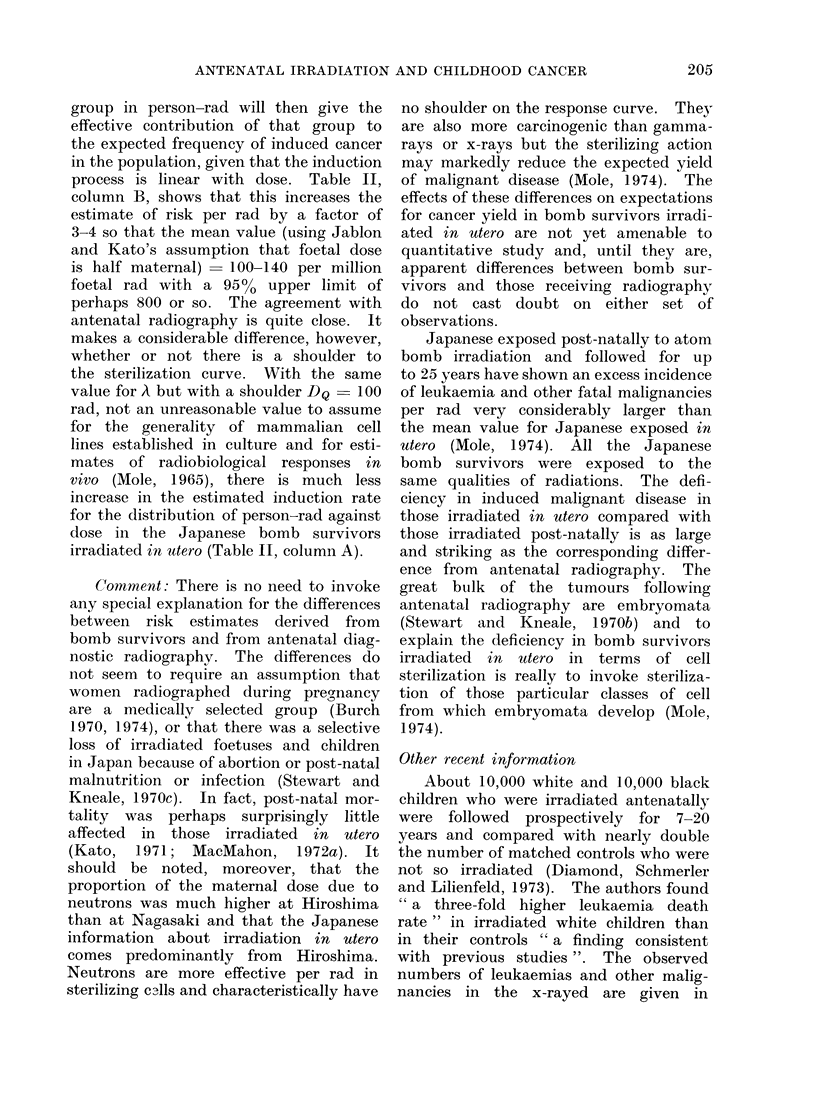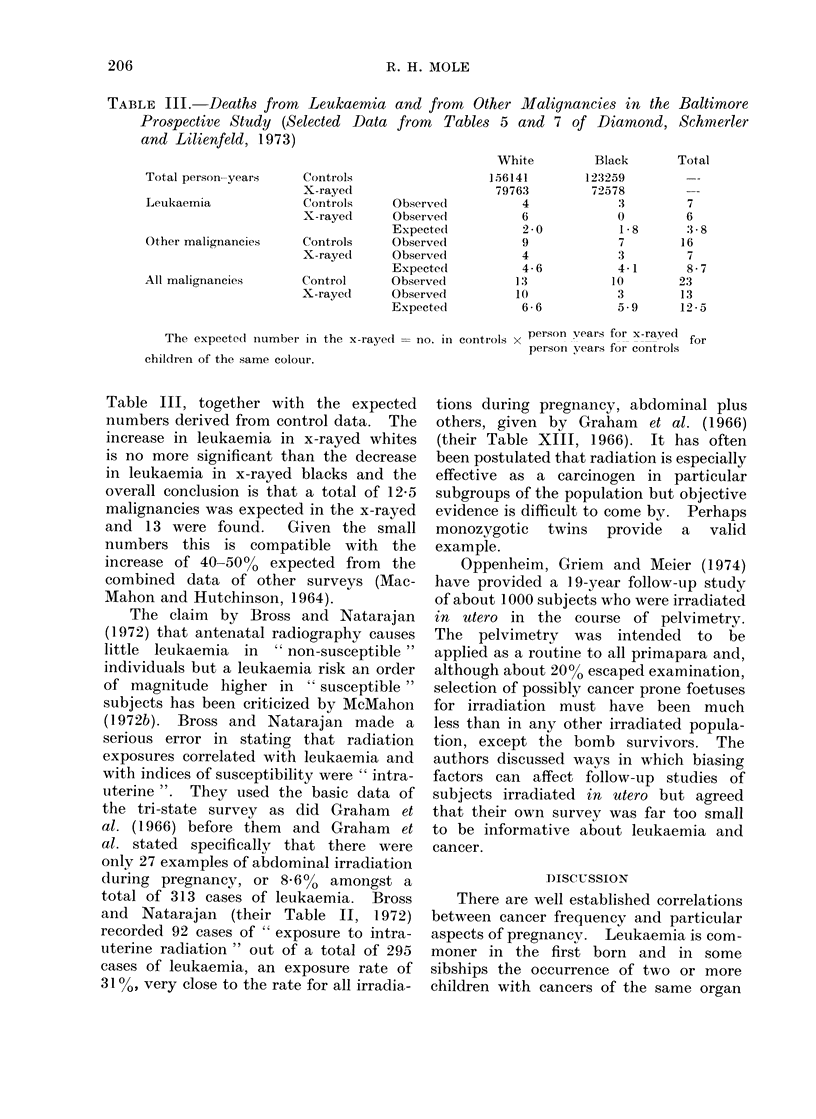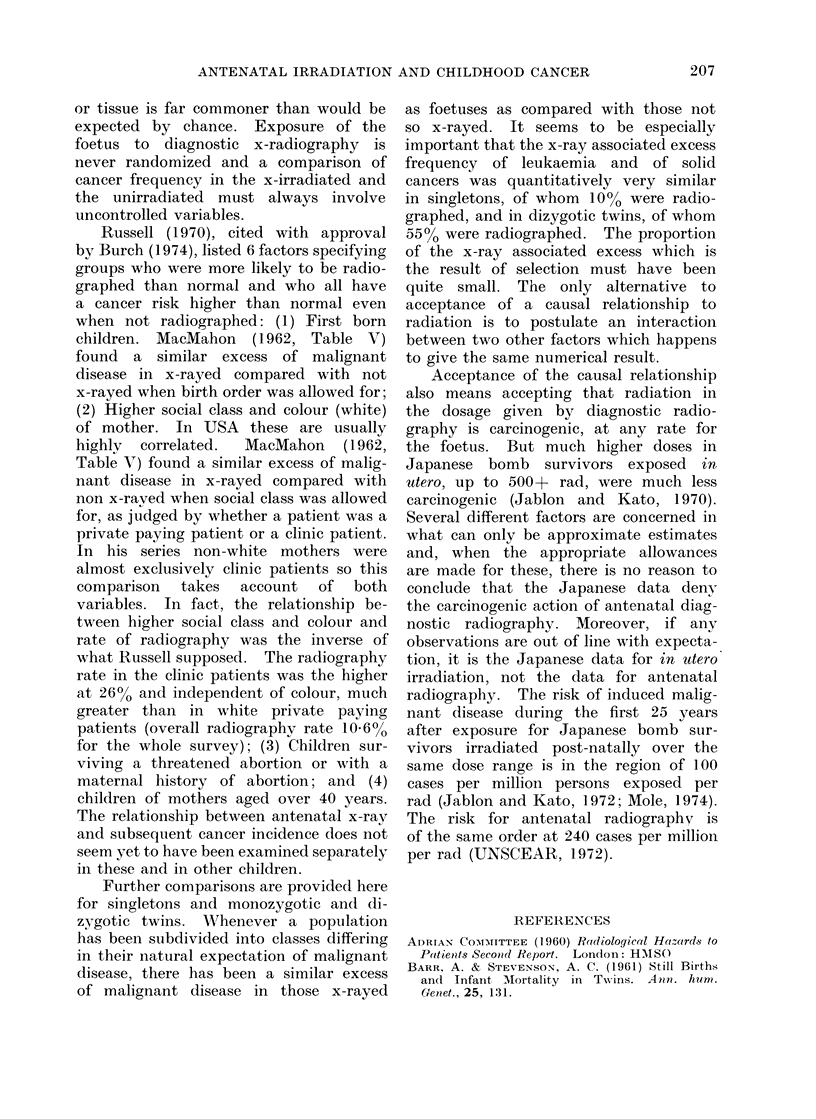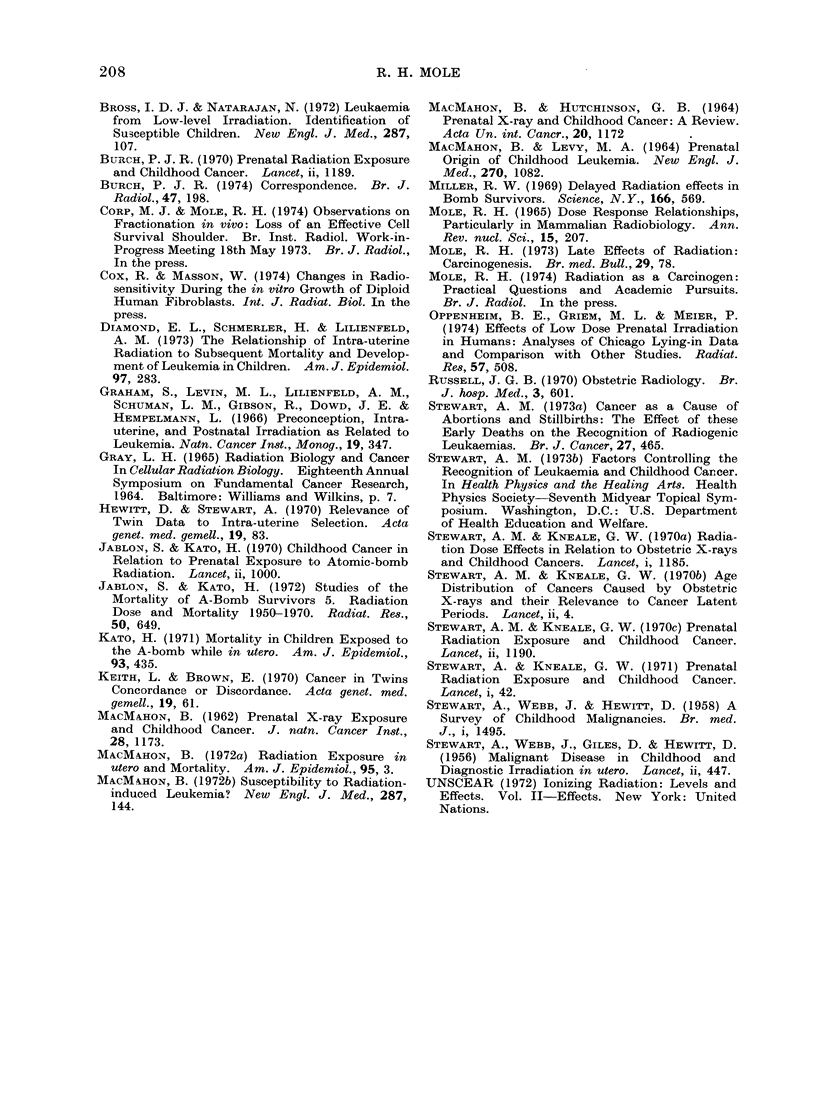# Antenatal Irradiation and Childhood Cancer: Causation or Coincidence?

**DOI:** 10.1038/bjc.1974.182

**Published:** 1974-09

**Authors:** R. H. Mole

## Abstract

A re-analysis of published data from the Oxford Childhood Cancer Survey shows that the frequency of leukaemia and of solid cancers in childhood is greater following antenatal x-radiography, not only in singleton births but also in monozygotic and dizygotic twins. The radiography rate was 10% in singletons and 55% in twins. A similar excess of leukaemia and of solid cancers in the x-rayed with such different rates of radiography is strong evidence for irradiation as the cause. The low observed frequency of malignant disease in Japanese bomb survivors exposed *in utero* may not be in serious conflict with this conclusion, as has been supposed.


*Br. J. Cancer* (1974) **30**, 199

### ANTENATAL IRRADIATION AND CHILDHOOD CANCER: CAUSATION OR COINCIDENCE?

R. H. MOLE

*From the Medical Research Council, Radiobiology Unit, Harwell, Didcot, Oxfordshire OX11 ORD*

Received 13 May 1974. Accepted 30 May 1974

**Summary.—A re-analysis of published data from the Oxford Childhood Cancer Survey shows that the frequency of leukaemia and of solid cancers in childhood is greater following antenatal x-radiography, not only in singleton births but also in monozygotic and dizygotic twins. The radiography rate was 10% in singletons and 55% in twins. A similar excess of leukaemia and of solid cancers in the x-rayed with such different rates of radiography is strong evidence for irradiation as the cause. The low observed frequency of malignant disease in Japanese bomb survivors exposed *in utero* may not be in serious conflict with this conclusion, as has been supposed.**

THE FIRST association between antenatal diagnostic x-irradiation and a subsequent increase in leukaemia and other childhood cancers was reported nearly 20 years ago (Stewart *et al.*, 1956; Stewart, Webb and Hewitt, 1958). A number of other surveys were made in the next few years, some confirming and some denying the association, but all of them in fact statistically compatible with the original finding of a 40–50% increase in deaths from malignant disease before the 10th birthday (MacMahon and Hutchinson, 1964). Even now, however, it can be claimed that there is no proof of a causal association and by inference or explicitly that the association is simply the consequence of a higher rate of diagnostic radiography in those foetuses predetermined to have a higher cancer rate anyway (Miller, 1969; Burch, 1970, 1974). Of course, such a hypothesis of coincidence does not of itself refute a hypothesis of causality or *vice versa*; both could be true. There have been two main observational problems. First, there seemed to be no comparable populations of foetuses with widely differing rates of exposure to x-rays which would allow the respective strengths of coincidence and causality to be compared. Secondly, Japanese bomb survivors irradiated *in utero* have shown only a very small excess of cancer, given certain assumptions far smaller than might be expected from the risk estimates derived from studies of antenatal diagnostic radiography (Jablon and Kato, 1970). It now seems possible to go some way to resolve both these problems and so to conclude that antenatal diagnostic radiography is truly carcinogenic, whether or not there is also a degree of selection for radiography of those with an above average cancer risk even when not radiographed.

### *Comparative observations on twin and singleton births*

Stewart (1973*a*, *b*) has published data on the numbers of fatal leukaemias and solid cancers in twins, of whom 55% were radiographed, and in singleton births, of whom 10% were radiographed. These allow calculation of death rates in the first 10 years of life (Table I). Twins of opposite sex are clearly dizygotic. It is commonly assumed with almost complete justification that there must

TABLE I.—*Leukaemias and other Malignancies in First* 10 *Years of Life for Singleton and Twin Births According to Antenatal x-ray Exposure* (*data of Stewart* 1973*a*, *b*)

| | Singletons* | Twins† | | | |
|---|---|---|---|---|---|
| | Births | All | Opposite sex (dizygotic) | Like sex | Mono-zygotic |
| Still birth rate (%) | 2·1 | 5·7 | 4·0 | 5·7 | 7·9 |
| Live born | 14771901 | 353114 | 127245 | 225869 | 98624 |
| Proportion x-rayed | 0·10 | 0·55 | 0·55 | 0·55 | 0·55 |
| Leukaemias: x-rayed | 511 | 51 | 17 | 33 | 16 |
| not x-rayed | 2955 | 19 | 9 | 9 | 0 |
| Solid cancers: x-rayed | 580 | 60 | 22 | 38 | 16 |
| not x-rayed | 3482 | 31 | 12 | 17 | 5 |
| *Rate per* 100000 | | | | | |
| Leukaemias: x-rayed | 34·6 | 26·3 | 24·3 | 26·6 | 29·5 |
| not x-rayed | 22·8 | 12·0 | 15·7 | 8·9 | nil |
| Solid cancers: x-rayed | 39·3 | 30·9 | 31·4 | 30·6 | 29·5 |
| not x-rayed | 26·9 | 19·5 | 21·0 | 16·7 | 11·3 |
| *Additional risk due to radiation per* 100000 | | | | | |
| Leukaemias | 11·8 | 14·3 | 9 | 18 | 29 |
| Solid cancers | 12·4 | 11·4 | 10 | 14 | 18 |
| *Relative risk in irradiated compared with unirradiated* | | | | | |
| Leukaemias | 1·5 | 2·2 | 1·5 | 3·0 | ++ |
| Solid cancers | 1·5 | 1·6 | 1·5 | 1·8 | 2·6 |

* The number and x-rayed proportion of singletons from Stewart (1973*a*). The numbers of leukaemias and solid cancers are given for singletons plus twins in Table III of Stewart (1973*b*) and subtraction of the twin data gives the numbers for singletons alone. The populations considered in Stewart (1973*a*, *b*) are identical (Stewart, 1974 personal communication).

† Twin data from Stewart (1973*a*). The radiography rate was very similar for like sex twins and opposite sex twins, with no significant differences either in the Oxford Childhood Cancer Survey or in the 1958 perinatal survey (Stewart, 1974, personal communication).

be a similar number of dizygotic twin births of like sex and this allows the number of monozygotic twin births to be derived by subtraction from the total of births of like sex. It may also be assumed that the deaths from malignant disease in dizygotic twins will be the same for like sex twins and for twins of opposite sex, so that the corresponding deaths for monozygotic twins can also be derived. The published data allow these calculations to be made separately for x-rayed and non x-rayed foetuses although there must be some small error because the sex of 2 of 70 twin leukaemias and of 2 of 91 twin cancers was not recorded (Stewart, 1973*a*); these therefore have to be omitted. Death of one twin *in utero* and survival of an apparent singleton will only reduce differences between singletons and twins (Hewitt and Stewart, 1970).

When singleton births and all twins combined are compared, Table I shows that non x-rayed singletons had a leukaemia rate almost twice that of non x-rayed twins and a rate for solid cancers also larger but less so. X-rayed singletons and twins each showed higher rates than the non x-rayed and the numerical increase associated with radiography was about the same for each type of birth and each class of malignant disease, in the range 11–14 cases per 100,000 radiographed. The proportionate increase was also similar, except for leukaemia in twins.

Separate analysis of presumed monozygotic and dizygotic twins (Table I) shows that the death rate from leukaemia and solid cancers in non x-rayed foetuses was smaller for each kind of twin than for singletons, but that dizygotics were much more like singletons than monozygotic twins. The excess of leukaemias and solid cancers in the x-rayed was similar in singletons and in dizygotic twins. The somewhat smaller excess of leukaemias and solid cancers in the latter is as might be expected because there is a larger proportion of single film examinations and therefore a somewhat smaller mean radiation dose for twins than singletons (cf. Stewart and Kneale, 1971). This factor makes the increase in leukaemias and solid cancers in x-rayed monozygotic twins even more noteworthy. The numerical increase associated with radiography was 2–3 times higher in monozygotic than in dizygotic twins (and the proportionate increase correspondingly greater).

*Comment:* Dizygotic twins will have the same genetic diversity as singleton births and their genetically determined predisposition to cancer would be expected to be broadly similar unless there is some influence of twinning *qua* twins. Their rates for leukaemia and for solid cancers were in fact similar, both when x-rayed and when not x-rayed. The only effect of twinning *per se* might be the 20–30% smaller rates in unirradiated dizygotic twins compared with unirradiated singletons. This could be associated with the greater perinatal mortality of such twins as compared with singletons (Table I) if Stewart's (1973*a*) hypothesis is accepted that malignant disease which kills in childhood is often initiated very early in development, and that foetuses and newborn infants who are incubating as yet unrecognized malignant disease are over-represented among perinatal deaths. A 20–30% loss, however, is not very important quantitatively in the present context. Moreover, it would affect the baseline, the predetermined cancer experience, to a similar degree in both the irradiated and unirradiated, and would thus not affect the numerical excess consequent on irradiation. A selective perinatal loss of twin cases of malignancies in the non x-rayed would indeed inflate the calculated ratios of observed to expected numbers in the x-rayed, and such ratios may therefore be less valuable indices of radiation effects than the simple numerical excess of cases in the irradiated above the unirradiated. The excess rate per 100,000 was the same within 20–25% for dizygotic twins and for singletons for both leukaemia and solid cancers, and the difference is of about the degree expected from the probable differences in average dose.

A comparison of singleton births, of whom 10% were irradiated, and dizygotic twins, of whom 55% were irradiated, should provide a critical test of the interpretation that all the excess malignant disease after irradiation *in utero* is the result of selection of the cancer prone for radiography. If this was the case. the excess cancer rate in x-rayed dizygotic twins should be much less than the excess in x-rayed singletons. Since the excess cancer rate per $10^5$ is so similar, 9–12 for leukaemia and 10–12 for solid cancers (Table I), the greater part of the excess must be attributed to causation, an excess of 2–3 being the maximum attributable to selection of the cancer prone. There was no difference in the frequency of radiography among surviving twins and those dying perinatally, 58·0% and 56·7% respectively, whereas the corresponding frequencies in singletons were 11% and 22·5% (data of 1958 perinatal mortality survey cited by Stewart, 1973*a*). It does not seem at all likely that specific perinatal and infant mortality in the irradiated reduced the observed death rate from malignant disease in childhood by a factor of 4–5 for singletons and not at all in dizygotic twins, the extent of the change required if the similarity in excess rates of leukaemia and of cancers in the x-rayed is to be attributed to selection of the cancer prone for radiography.

Monozygotic twins differ from dizygotic twins and singletons in many respects. Their still birth rate and infant mortality rate is far higher (Barr and Stevenson, 1961) and in the non x-rayed the death rate in childhood from leukaemia and from solid cancers is clearly smaller (Table I). The complete absence of leukaemias may be an artefact of small numbers. Hewitt and Stewart (1970) wrote that " the deficit of like sex pairs not x-rayed implies selective elimination of twin zygotes from neoplasms the result of cell damage incurred at or shortly after conception ". Table I shows that the deficit in like sex twins is very largely the consequence of a much larger deficit in monozygotic twins and, if so, this suggestion as to cause must apply in much greater degree to monozygotic than to dizygotic twins and cannot be due simply to twinning. An alternative explanation which does not invoke " cell damage " at or shortly after conception, is specific to monozygotic twins and particularly applicable to leukaemia. If leukaemia originated in one twin, it would have a high chance of affecting the other twin as a consequence of migration of cells from the one monozygotic twin through a common chorion into a perfectly histocompatible host, the other twin (cf. MacMahon and Levy, 1964). In fact, there seems to be a very high frequency of concordance between monozygotic twins dying of leukaemia in the first few years of life (MacMahon and Levy, 1964; Keith and Brown, 1970). Whatever the mechanism underlying this concordance, there must be severe selection pressure against a simultaneous genetic predisposition to " spontaneous " leukaemia and to monozygotic twinning, and this would lead to a low natural leukaemia rate in unirradiated monozygotic twins. The same argument might even account for such twins having a lower frequency of solid cancers: in the embryo, if not in the foetus, some migration of tumour cells from one twin into the other is not an unreasonable thing to imagine. It would be interesting to learn the facts about the numbers of concordant cancers in pairs of monozygotic twins separately (cf. Stewart, 1973*b*).

There is no more selection for radiography of monozygotic than of dizygotic twins and effects of twinning *qua* twins must be the same. Thus, it is noteworthy that, as judged by the numerical excess of cases, the sensitivity to radiation induction of malignant disease seemed to be 2–3 times larger in monozygotic twins than in dizygotic twins. There is no definite explanation: irradiation is most frequent in the third trimester and migration of cells from one twin to another may seem increasingly unlikely as pregnancy progresses, and would also imply that much of the radiation induced excess in monozygotic twins would be attributable to the occurrence of twin pairs carrying the same type of cancer. If so, there would be no need to postulate genetically determined differences between monozygotic twins and others in susceptibility to carcinogenesis by ionizing radiation. Whether or not this is so, the data on monozygotic twins show an inverse correlation between natural and radiation induced frequency of malignant disease, not the positive correlation expected if the association of radiography and increased cancer frequency was the result of selection of the cancer prone for radiography, and thus confirm the causal relationship between antenatal exposure to irradiation and a subsequent increase in malignant disease.

### *Japanese bomb survivors irradiated* in utero

These are a special subgroup in the long term follow-up of the survivors at Hiroshima and Nagasaki. Each survivor has provided a detailed statement of exactly where he or she was at the time of the atom bomb explosions and this, taken in conjunction with detailed and elaborate studies of the radiations produced by the bombs and of the modifications in flux according to the scattering and shielding by air and by building materials of various kinds, has enabled a reasonably accurate estimate to be made of the radiation dose for each individual survivor, although there is a minority of cases where this has so far proved impossible. Jablon and Kato (1970) and Kato (1971) provided the data on cancer deaths over the period 0–24 years after exposure on survivors irradiated *in utero* classified according to maternal dose.

The frequency of induced malignancy might be expected to increase with increasing radiation dose and a linear relationship is commonly postulated. Given these assumptions Jablon and Kato (1970) showed that there were far fewer tumours after antenatal irradiation in bomb survivors than would be expected from the estimates of risk per rad of diagnostic x-rays provided by Stewart and Kneale (1970*a*). Jablon and Kato's (1970) argument was as follows: The whole population of Japanese bomb survivors irradiated *in utero* included 1 cancer death between 0 and 10 years of age (Table II). The number of cancers expected from national statistics for those receiving 1–499 rad (maternal dose) = 0·40 so that the excess = 0·60. The upper 95% confidence limit of 1 case = 4·74 cases, making the corresponding limit for the excess 4·34 cases. The total maternal rad = 34,933 (but see footnote (e) Table II) and the corresponding values for the mean and for the upper limit of the risk per million maternal rad are then 17 and 124 respectively. The foetal dose will be smaller than the maternal because of attenuation of radiation in the body of the mother, a reduction at most by a factor of 2, which will raise these values to at most 34 and 248. The only numerical estimate of the risk for antenatal diagnostic radiography available to

TABLE II.—*Japanese Atom Bomb Survivors Irradiated* in utero*: Radiation Doses, Number of Subjects and Number of Deaths from Malignant Disease*[a]*, and Calculation of Risk Coefficient per Rad on the Linear Hypothesis for Carcinogenesis According to Various Postulated Hypotheses for Retention of Reproductive Integrity*

| Dose rad | No. of cases exposed *in utero* | Deaths from malignant disease 0–10 years old: Observed | Expected | Maternal-rad (thousands) | Postulated fractional retention of reproductive integrity: A[b] | B[c] |
|---|---|---|---|---|---|---|
| <1 | 551 | 0 | 0·32 | nil | — | — |
| 1–9 | 244 | 0 | 0·14 } | 5·5 | 1 | 0·8–0·9 |
| 10–39 | 223 | 0 | 0·13 } | | | |
| 40–179 | 180 | 1 | 0·10 | 14·3[d] | 0·7 | 0·3–0·4 |
| 180–299 | 35 | 0 | 0·02 | 8·4[d] | 0·3 | 0·05–0·1 |
| 300–499 | 17 | 0 | 0·01 | 6·7 | 0·05 | 0·01–0·02 |
| 500+ | 16 | 0 | 0·01 | 29·6 | small | small |
| Unknown | 26 | 0 | 0·01 | — | | |
| Carcinogenically effective maternal-rad (thousands) on linear induction hypothesis | | | | 34·9[e] | 18 | 9–12 |
| Corresponding induction rate per million per rad[f] | | | | 17 | 30 | 50—70 |

[a] Data derived from Table I of Jablon and Kato (1970) and Table II of Kato (1971).

[b] A type C sterilization curve with shoulder $N = N_0[1 - (1 - e^{-\lambda D})^n]$ with $\lambda = 0{\cdot}01$ and $n = 2{\cdot}7$ so that $D_Q = 100$ rad.

[c] B strictly exponential sterilization curve $N = N_0 e^{-\lambda D}$ with $\lambda = 0{\cdot}01$–$0{\cdot}013$ (cf. Cox and Masson, 1974).

[d] Estimated by author from the summed value of 22·7 Krad for the dose range 40–299 rad.

[e] Jablon and Kato (1970) excluded from consideration "the 16 cases with dose estimates exceeding 500 rad, which seem suspect".

[f] Derived by dividing the excess deaths from malignant disease (observed–expected) by the carcinogenically effective maternal-rad = $\Sigma$ (maternal-rad × fractional retention of reproductive integrity).

Jablon and Kato was 572 per million foetal rad, 95% range 300–800 (Stewart and Kneale, 1970*a*). Thus, the two sets of observations seemed irreconcilable.

However, the estimate of risk for antenatal diagnostic radiography depends on assessments of the mean dose per examination, which can vary several-fold. The actual dose received by an individual foetus in the course of pelvimetry varied by more than 2 orders of magnitude in U.K. in 1957 (Adrian Committee, 1960, Fig. 5F), and there are other uncertainties relating to changes in radiographic technique over a period of 20 years. Stewart and Kneale (1970*c*) felt that 356 and 291 were as good mean values of risk as 572 and for the same data UNSCEAR (1972) chose the value of 240 (with a 95% range persumably of the order of 120–360 per million per rad). The upper limit of the risk derived from Japanese data for bomb irradiation can then be regarded as compatible with the estimates for diagnostic radiography, but only if all the assumptions in the calculations are chosen with that intent.

On the other hand, the basic assumptions underlying these comparisons may be in error. It is not in fact the case that increasing radiation dose always leads to an increased frequency of induced malignancy (Mole, 1973). It is the general rule, both in experimental work and in observations on man, that there is an optimum radiation dose and that for larger exposures the frequency of induction per unit dose decreases. There is an elementary radiobiological reason for this. Whatever the mechanism of cancer induction, there must be a multiplication of the transformed cell or cells before the cancer can become clinically evident. Ionizing radiation can kill cells and can sterilize them so that, though viable for a time, they cannot reproduce. Thus, the observed frequency of induced malignant disease after doses in the cell sterilizing range will always be less than the frequency expected from the induction process. The deficiency will increase with dose in proportion to the increasing probability of sterilization of cells which would otherwise have been able to divide and so form a "tumour". The idea was first applied quantitatively by Gray (1965) and has proved applicable to other experimental data and to observations on cancer frequencies in irradiated human populations (Mole, 1974). When applied to the data on antenatal exposure of human foetuses, the discrepancies outlined above are reduced to acceptable levels.

Cell sterilization by ionizing radiation is exponentially related to radiation dose

$$N = N_0 e^{-\lambda D}$$

where

$N_0$ = initial number of cells
$N$ = number of cells surviving with maintained reproductive integrity
$\lambda$ = a constant which may be characteristic of the cells concerned
$D$ = radiation dose.

With x-rays and $\gamma$-rays *in vitro* there is commonly a shoulder on the relationship so that for doses from $D = 0$ to about $D = D_Q$ the response is less than exponential. However, *in vivo* observations suggest that the presence or absence of a shoulder in haemopoietic tissue depends on the level of cellular activity (Corp and Mole, 1974) and the only available data on freshly isolated human foetal cells (as distinct from the established mammalian cell lines on which almost all other radiobiological work has been done) have shown a strictly exponential response without shoulder (Cox and Masson, 1974).

The data on numbers of cases and maternal dose for bomb survivors irradiated *in utero* are given in Table II together with estimates of the fraction of cells surviving with maintained reproductive integrity using the value for $\lambda$ found directly for human foetal cells by Cox and Masson (1974). The surviving fraction for a given dose group multiplied by the total radiation exposure of that group in person–rad will then give the effective contribution of that group to the expected frequency of induced cancer in the population, given that the induction process is linear with dose. Table II, column B, shows that this increases the estimate of risk per rad by a factor of 3–4 so that the mean value (using Jablon and Kato's assumption that foetal dose is half maternal) = 100–140 per million foetal rad with a 95% upper limit of perhaps 800 or so. The agreement with antenatal radiography is quite close. It makes a considerable difference, however, whether or not there is a shoulder to the sterilization curve. With the same value for $\lambda$ but with a shoulder $D_Q = 100$ rad, not an unreasonable value to assume for the generality of mammalian cell lines established in culture and for estimates of radiobiological responses *in vivo* (Mole, 1965), there is much less increase in the estimated induction rate for the distribution of person–rad against dose in the Japanese bomb survivors irradiated *in utero* (Table II, column A).

*Comment:* There is no need to invoke any special explanation for the differences between risk estimates derived from bomb survivors and from antenatal diagnostic radiography. The differences do not seem to require an assumption that women radiographed during pregnancy are a medically selected group (Burch 1970, 1974), or that there was a selective loss of irradiated foetuses and children in Japan because of abortion or post-natal malnutrition or infection (Stewart and Kneale, 1970*c*). In fact, post-natal mortality was perhaps surprisingly little affected in those irradiated *in utero* (Kato, 1971; MacMahon, 1972*a*). It should be noted, moreover, that the proportion of the maternal dose due to neutrons was much higher at Hiroshima than at Nagasaki and that the Japanese information about irradiation *in utero* comes predominantly from Hiroshima. Neutrons are more effective per rad in sterilizing cells and characteristically have no shoulder on the response curve. They are also more carcinogenic than gamma-rays or x-rays but the sterilizing action may markedly reduce the expected yield of malignant disease (Mole, 1974). The effects of these differences on expectations for cancer yield in bomb survivors irradiated *in utero* are not yet amenable to quantitative study and, until they are, apparent differences between bomb survivors and those receiving radiography do not cast doubt on either set of observations.

Japanese exposed post-natally to atom bomb irradiation and followed for up to 25 years have shown an excess incidence of leukaemia and other fatal malignancies per rad very considerably larger than the mean value for Japanese exposed *in utero* (Mole, 1974). All the Japanese bomb survivors were exposed to the same qualities of radiations. The deficiency in induced malignant disease in those irradiated *in utero* compared with those irradiated post-natally is as large and striking as the corresponding difference from antenatal radiography. The great bulk of the tumours following antenatal radiography are embryomata (Stewart and Kneale, 1970*b*) and to explain the deficiency in bomb survivors irradiated *in utero* in terms of cell sterilization is really to invoke sterilization of those particular classes of cell from which embryomata develop (Mole, 1974).

*Other recent information*

About 10,000 white and 10,000 black children who were irradiated antenatally were followed prospectively for 7–20 years and compared with nearly double the number of matched controls who were not so irradiated (Diamond, Schmerler and Lilienfeld, 1973). The authors found " a three-fold higher leukaemia death rate " in irradiated white children than in their controls " a finding consistent with previous studies ". The observed numbers of leukaemias and other malignancies in the x-rayed are given in

TABLE III.—*Deaths from Leukaemia and from Other Malignancies in the Baltimore Prospective Study (Selected Data from Tables 5 and 7 of Diamond, Schmerler and Lilienfeld*, 1973)

| | | | White | Black | Total |
|---|---|---|---|---|---|
| Total person–years | Controls | | 156141 | 123259 | — |
| | X-rayed | | 79763 | 72578 | — |
| Leukaemia | Controls | Observed | 4 | 3 | 7 |
| | X-rayed | Observed | 6 | 0 | 6 |
| | | Expected | 2·0 | 1·8 | 3·8 |
| Other malignancies | Controls | Observed | 9 | 7 | 16 |
| | X-rayed | Observed | 4 | 3 | 7 |
| | | Expected | 4·6 | 4·1 | 8·7 |
| All malignancies | Control | Observed | 13 | 10 | 23 |
| | X-rayed | Observed | 10 | 3 | 13 |
| | | Expected | 6·6 | 5·9 | 12·5 |

The expected number in the x-rayed = no. in controls $\times \frac{\text{person years for x-rayed}}{\text{person years for controls}}$ for children of the same colour.

Table III, together with the expected numbers derived from control data. The increase in leukaemia in x-rayed whites is no more significant than the decrease in leukaemia in x-rayed blacks and the overall conclusion is that a total of 12·5 malignancies was expected in the x-rayed and 13 were found. Given the small numbers this is compatible with the increase of 40–50% expected from the combined data of other surveys (MacMahon and Hutchinson, 1964).

The claim by Bross and Natarajan (1972) that antenatal radiography causes little leukaemia in " non-susceptible " individuals but a leukaemia risk an order of magnitude higher in " susceptible " subjects has been criticized by McMahon (1972*b*). Bross and Natarajan made a serious error in stating that radiation exposures correlated with leukaemia and with indices of susceptibility were " intrauterine ". They used the basic data of the tri-state survey as did Graham *et al.* (1966) before them and Graham *et al.* stated specifically that there were only 27 examples of abdominal irradiation during pregnancy, or 8·6% amongst a total of 313 cases of leukaemia. Bross and Natarajan (their Table II, 1972) recorded 92 cases of " exposure to intrauterine radiation " out of a total of 295 cases of leukaemia, an exposure rate of 31%, very close to the rate for all irradiations during pregnancy, abdominal plus others, given by Graham *et al.* (1966) (their Table XIII, 1966). It has often been postulated that radiation is especially effective as a carcinogen in particular subgroups of the population but objective evidence is difficult to come by. Perhaps monozygotic twins provide a valid example.

Oppenheim, Griem and Meier (1974) have provided a 19-year follow-up study of about 1000 subjects who were irradiated *in utero* in the course of pelvimetry. The pelvimetry was intended to be applied as a routine to all primapara and, although about 20% escaped examination, selection of possibly cancer prone foetuses for irradiation must have been much less than in any other irradiated population, except the bomb survivors. The authors discussed ways in which biasing factors can affect follow-up studies of subjects irradiated *in utero* but agreed that their own survey was far too small to be informative about leukaemia and cancer.

### DISCUSSION

There are well established correlations between cancer frequency and particular aspects of pregnancy. Leukaemia is commoner in the first born and in some sibships the occurrence of two or more children with cancers of the same organ or tissue is far commoner than would be expected by chance. Exposure of the foetus to diagnostic x-radiography is never randomized and a comparison of cancer frequency in the x-irradiated and the unirradiated must always involve uncontrolled variables.

Russell (1970), cited with approval by Burch (1974), listed 6 factors specifying groups who were more likely to be radiographed than normal and who all have a cancer risk higher than normal even when not radiographed: (1) First born children. MacMahon (1962, Table V) found a similar excess of malignant disease in x-rayed compared with not x-rayed when birth order was allowed for; (2) Higher social class and colour (white) of mother. In USA these are usually highly correlated. MacMahon (1962, Table V) found a similar excess of malignant disease in x-rayed compared with non x-rayed when social class was allowed for, as judged by whether a patient was a private paying patient or a clinic patient. In his series non-white mothers were almost exclusively clinic patients so this comparison takes account of both variables. In fact, the relationship between higher social class and colour and rate of radiography was the inverse of what Russell supposed. The radiography rate in the clinic patients was the higher at 26% and independent of colour, much greater than in white private paying patients (overall radiography rate 10·6% for the whole survey); (3) Children surviving a threatened abortion or with a maternal history of abortion; and (4) children of mothers aged over 40 years. The relationship between antenatal x-ray and subsequent cancer incidence does not seem yet to have been examined separately in these and in other children.

Further comparisons are provided here for singletons and monozygotic and dizygotic twins. Whenever a population has been subdivided into classes differing in their natural expectation of malignant disease, there has been a similar excess of malignant disease in those x-rayed as foetuses as compared with those not so x-rayed. It seems to be especially important that the x-ray associated excess frequency of leukaemia and of solid cancers was quantitatively very similar in singletons, of whom 10% were radiographed, and in dizygotic twins, of whom 55% were radiographed. The proportion of the x-ray associated excess which is the result of selection must have been quite small. The only alternative to acceptance of a causal relationship to radiation is to postulate an interaction between two other factors which happens to give the same numerical result.

Acceptance of the causal relationship also means accepting that radiation in the dosage given by diagnostic radiography is carcinogenic, at any rate for the foetus. But much higher doses in Japanese bomb survivors exposed *in utero*, up to 500+ rad, were much less carcinogenic (Jablon and Kato, 1970). Several different factors are concerned in what can only be approximate estimates and, when the appropriate allowances are made for these, there is no reason to conclude that the Japanese data deny the carcinogenic action of antenatal diagnostic radiography. Moreover, if any observations are out of line with expectation, it is the Japanese data for *in utero* irradiation, not the data for antenatal radiography. The risk of induced malignant disease during the first 25 years after exposure for Japanese bomb survivors irradiated post-natally over the same dose range is in the region of 100 cases per million persons exposed per rad (Jablon and Kato, 1972; Mole, 1974). The risk for antenatal radiography is of the same order at 240 cases per million per rad (UNSCEAR, 1972).

### REFERENCES

ADRIAN COMMITTEE (1960) *Radiological Hazards to Patients Second Report.* London: HMSO

BARR, A. & STEVENSON, A. C. (1961) Still Births and Infant Mortality in Twins. *Ann. hum. Genet.*, **25**, 131.

BROSS, I. D. J. & NATARAJAN, N. (1972) Leukaemia from Low-level Irradiation. Identification of Susceptible Children. *New Engl. J. Med.*, **287**, 107.

BURCH, P. J. R. (1970) Prenatal Radiation Exposure and Childhood Cancer. *Lancet*, ii, 1189.

BURCH, P. J. R. (1974) Correspondence. *Br. J. Radiol.*, **47**, 198.

CORP, M. J. & MOLE, R. H. (1974) Observations on Fractionation *in vivo*: Loss of an Effective Cell Survival Shoulder. Br. Inst. Radiol. Work-in-Progress Meeting 18th May 1973. *Br. J. Radiol.*, In the press.

COX, R. & MASSON, W. (1974) Changes in Radiosensitivity During the *in vitro* Growth of Diploid Human Fibroblasts. *Int. J. Radiat. Biol.* In the press.

DIAMOND, E. L., SCHMERLER, H. & LILIENFELD, A. M. (1973) The Relationship of Intra-uterine Radiation to Subsequent Mortality and Development of Leukemia in Children. *Am. J. Epidemiol.* **97**, 283.

GRAHAM, S., LEVIN, M. L., LILIENFELD, A. M., SCHUMAN, L. M., GIBSON, R., DOWD, J. E. & HEMPELMANN, L. (1966) Preconception, Intrauterine, and Postnatal Irradiation as Related to Leukemia. *Natn. Cancer Inst., Monog.*, **19**, 347.

GRAY, L. H. (1965) Radiation Biology and Cancer In *Cellular Radiation Biology*. Eighteenth Annual Symposium on Fundamental Cancer Research, 1964. Baltimore: Williams and Wilkins, p. 7.

HEWITT, D. & STEWART, A. (1970) Relevance of Twin Data to Intra-uterine Selection. *Acta genet. med. gemell.*, **19**, 83.

JABLON, S. & KATO, H. (1970) Childhood Cancer in Relation to Prenatal Exposure to Atomic-bomb Radiation. *Lancet*, ii, 1000.

JABLON, S. & KATO, H. (1972) Studies of the Mortality of A-Bomb Survivors 5. Radiation Dose and Mortality 1950–1970. *Radiat. Res.*, **50**, 649.

KATO, H. (1971) Mortality in Children Exposed to the A-bomb while *in utero*. *Am. J. Epidemiol.*, **93**, 435.

KEITH, L. & BROWN, E. (1970) Cancer in Twins Concordance or Discordance. *Acta genet. med. gemell.*, **19**, 61.

MACMAHON, B. (1962) Prenatal X-ray Exposure and Childhood Cancer. *J. natn. Cancer Inst.*, **28**, 1173.

MACMAHON, B. (1972*a*) Radiation Exposure *in utero* and Mortality. *Am. J. Epidemiol.*, **95**, 3.

MACMAHON, B. (1972*b*) Susceptibility to Radiation-induced Leukemia? *New Engl. J. Med.*, **287**, 144.

MACMAHON, B. & HUTCHINSON, G. B. (1964) Prenatal X-ray and Childhood Cancer: A Review. *Acta Un. int. Cancr.*, **20**, 1172 .

MACMAHON, B. & LEVY, M. A. (1964) Prenatal Origin of Childhood Leukemia. *New Engl. J. Med.*, **270**, 1082.

MILLER, R. W. (1969) Delayed Radiation effects in Bomb Survivors. *Science, N.Y.*, **166**, 569.

MOLE, R. H. (1965) Dose Response Relationships, Particularly in Mammalian Radiobiology. *Ann. Rev. nucl. Sci.*, **15**, 207.

MOLE, R. H. (1973) Late Effects of Radiation: Carcinogenesis. *Br. med. Bull.*, **29**, 78.

MOLE, R. H. (1974) Radiation as a Carcinogen: Practical Questions and Academic Pursuits. *Br. J. Radiol.* In the press.

OPPENHEIM, B. E., GRIEM, M. L. & MEIER, P. (1974) Effects of Low Dose Prenatal Irradiation in Humans: Analyses of Chicago Lying-in Data and Comparison with Other Studies. *Radiat. Res*, **57**, 508.

RUSSELL, J. G. B. (1970) Obstetric Radiology. *Br. J. hosp. Med.*, **3**, 601.

STEWART, A. M. (1973*a*) Cancer as a Cause of Abortions and Stillbirths: The Effect of these Early Deaths on the Recognition of Radiogenic Leukaemias. *Br. J. Cancer*, **27**, 465.

STEWART, A. M. (1973*b*) Factors Controlling the Recognition of Leukaemia and Childhood Cancer. In *Health Physics and the Healing Arts*. Health Physics Society—Seventh Midyear Topical Symposium. Washington, D.C.: U.S. Department of Health Education and Welfare.

STEWART, A. M. & KNEALE, G. W. (1970*a*) Radiation Dose Effects in Relation to Obstetric X-rays and Childhood Cancers. *Lancet*, i, 1185.

STEWART, A. M. & KNEALE, G. W. (1970*b*) Age Distribution of Cancers Caused by Obstetric X-rays and their Relevance to Cancer Latent Periods. *Lancet*, ii, 4.

STEWART, A. M. & KNEALE, G. W. (1970*c*) Prenatal Radiation Exposure and Childhood Cancer. *Lancet*, ii, 1190.

STEWART, A. & KNEALE, G. W. (1971) Prenatal Radiation Exposure and Childhood Cancer. *Lancet*, i, 42.

STEWART, A., WEBB, J. & HEWITT, D. (1958) A Survey of Childhood Malignancies. *Br. med. J.*, i, 1495.

STEWART, A., WEBB, J., GILES, D. & HEWITT, D. (1956) Malignant Disease in Childhood and Diagnostic Irradiation *in utero*. *Lancet*, ii, 447.

UNSCEAR (1972) Ionizing Radiation: Levels and Effects. Vol. II—Effects. New York: United Nations.